# Predicted breeding values for relative scrapie susceptibility for genotyped and ungenotyped sheep

**DOI:** 10.1186/s12711-024-00947-x

**Published:** 2024-12-18

**Authors:** Jón H. Eiríksson, Þórdís Þórarinsdóttir, Egill Gautason

**Affiliations:** 1 Faculty of Agricultural Sciences, Agricultural University of Iceland, 311 Borgarnes, Iceland; 2 The Icelandic Agricultural Advisory Centre, Óseyri 2, 603 Akureyri, Iceland

## Abstract

**Background:**

Scrapie is an infectious prion disease in sheep. Selective breeding for resistant genotypes of the prion protein gene (*PRNP*) is an effective way to prevent scrapie outbreaks. Genotyping all selection candidates in a population is expensive but existing pedigree records can help infer the probabilities of genotypes in relatives of genotyped animals.

**Results:**

We used linear models to predict allele content for the various *PRNP* alleles found in Icelandic sheep and compiled the available estimates of relative scrapie susceptibility (RSS) associated with *PRNP* genotypes from the literature. Using the predicted allele content and the genotypic RSS we calculated estimated breeding values (EBV) for RSS. We tested the predictions on simulated data under different scenarios that varied in the proportion of genotyped sheep, genotyping strategy, pedigree recording accuracy, genotyping error rates and assumed heritability of allele content. Prediction of allele content for rare alleles was less successful than for alleles with moderate frequencies. The accuracy of allele content and RSS EBV predictions was not affected by the assumed heritability, but the dispersion of prediction was affected. In a scenario where 40% of rams were genotyped and no errors in genotyping or recorded pedigree, the accuracy of RSS EBV for ungenotyped selection candidates was 0.49. If only 20% of rams were genotyped, or rams and ewes were genotyped randomly, or there were 10% pedigree errors, or there were 2% genotyping errors, the accuracy decreased by 0.07, 0.08, 0.03 and 0.04, respectively. With empirical data, the accuracy of RSS EBV for ungenotyped sheep was 0.46–0.65.

**Conclusions:**

A linear model for predicting allele content for the *PRNP* gene, combined with estimates of relative susceptibility associated with *PRNP* genotypes, can provide RSS EBV for scrapie resistance for ungenotyped selection candidates with accuracy up to 0.65. These RSS EBV can complement selection strategies based on *PRNP* genotypes, especially in populations where resistant genotypes are rare.

**Supplementary Information:**

The online version contains supplementary material available at 10.1186/s12711-024-00947-x.

## Background

Scrapie is a fatal, infectious, degenerative prion disease affecting the nervous system of sheep and goats. The susceptibility of sheep to scrapie is largely dependent on the genotype of the prion protein gene (*PRNP*). In Europe, polymorphisms in codons 136, 154, and 171 of the reading frame of the *PRNP* are common and lead to varying differences in susceptibility to scrapie [1]. Based on the amino acids coded at these three codons, the most common alleles of *PRNP* are denoted as ARR, AHQ, ARQ and VRQ, in order from the least susceptible (most resistant) to the most susceptible (least resistant) to scrapie [1, 2].

Icelandic sheep are an example of a breed where the ARR allele, associated with scrapie resistance, appears in a very low frequency [3]. However, the AHQ allele, associated with partial scrapie resistance [2, 4], has a frequency of around 8% [5]. Additionally, polymorphisms at codons 137, 138 and 151 of *PRNP* are present in the Icelandic population [3, 4]. Sheep carrying genotypes that codes for threonine (T) in codon 137 instead of the wild-type methionine, here denoted T137, show scrapie resistance in the Sarda sheep breed in Italy [6, 7]. Furthermore, results from Icelandic sheep suggest potential resistance linked to cysteine at codon 151, denoted herein as C151 [4, 8].

Genetic selection for resistant genotypes is an effective method for scrapie control [9, 10]. While *PRNP* genotyping is widely available, genotyping every selection candidate is expensive. However, recorded pedigree allows genotype prediction for ungenotyped relatives of genotyped animals, thus reducing cost while selecting for *PRNP* genotypes. As an example, Sawalha et al. [11] used segregation analysis to predict the genotype at codon 171 in *PRNP*, yielding probabilities for the three possible genotypes. However, when multiple alleles are considered, selection decisions based on such predictions could become very complicated.

For large livestock pedigrees, linear models that treat allele content (i.e. number of copies of an allele that an individual carries) as traits are a simple and efficient way for predicting genotypes in ungenotyped animals [12, 13]. Boareki et al. [14] tried a linear model to predict scrapie resistance based on *PRNP* genotypes. However, their model considered the allele content for each allele independently in single trait models, ignoring that the presence of one allele indicates absence of another [15]. Further, Boareki et al. [14] assigned numerical values to *PRNP* genotypes for scrapie resistance based on 5 risk groups. Each group consisted of multiple genotypes, some with differences in susceptibilities as shown in previous studies [2, 4]. Multiple studies have compared risk of scrapie in sheep carrying different *PRNP* genotypes [2, 4, 9], thus providing odds-ratios or other estimates for the relative susceptibility to scrapie (RSS) associated with the *PRNP* genotypes. The estimated RSS of genotypes might offer more accurate numerical estimates of scrapie susceptibility associated with the different alleles of *PRNP* than risk groups.

Although, in theory, allele content is completely inherited, linear model applications typically assume a heritability of less than 1.0, e.g. 0.99 [12]. Further, errors in genotyping or pedigree recording introduce random discrepancies, which in turn result in a lower perceived heritability. Forneris et al. [16] proposed using heritability estimates as quality control metrics for individual single nucleotide polymorphisms (SNPs), with reduced heritability indicating frequent genotyping errors.

The objectives of this study were to (1) assess the accuracy of a multi-trait linear model in predicting the allele content of ungenotyped animals for the multi-allelic *PRNP*, (2) compile existing estimates of the RSS associated with different *PRNP* genotypes, and (3) present calculations of estimated breeding value (EBV) of RSS based on the predicted allele content. Both simulated and empirical data from the Icelandic sheep population were used to assess the methodologies.

## Methods

In this section, first, we provide our definition of the breeding value for scrapie susceptibility, measured as RSS. Second, we outline the method for predicting the allele content for a single locus with multiple alleles. Third, we describe the methods employed to determine the level of RSS associated with each genotype. Fourth, we outline the assessment of the accuracy of the predicted allele content and EBV based on simulated and empirical data.

### Breeding values for scrapie resistance

The prevalence of scrapie in affected flocks depends on multiple factors in addition to the genotype of the sheep, such as environmental factors, scrapie strains and time from the onset of the scrapie outbreak [17]. Therefore, direct scrapie risk estimates across studies are not consistently meaningful. However, we made the simplifying assumption that the association of RSS with each genotype is constant across environments, scrapie strains and stages of scrapie outbreak. We quantified RSS as the odds-ratio of each genotype compared with the wildtype genotype ARQ/ARQ. We denote the RSS of the genotype consisting of alleles $k$ and $l$ as ${g}_{k,l}$. These assumptions enabled us to define breeding values for RSS and use results from multiple studies for evaluating RSS.

Breeding values of an individual are defined based on the mean allele effects (${\alpha }_{i}$) of the individual’s alleles. In the presence of dominance effects, mean allele effects depend on the allele frequency in the population in addition to the additive and dominance effects of the allele [18]. In a multi-allelic locus, generalising the bi-allelic formulas of Falconer and Mackay [18], the mean allele effect of allele $i$ within a population can be calculated as:

1 $$\alpha_{i} \, = \,\sum\nolimits_{l} {p_{l} \left( {g_{i,l} } \right)} \, - \,\sum\nolimits_{k} {\sum\nolimits_{l} {p_{k} p_{l} \left( {g_{k,l} } \right)} } \, = \,\sum\nolimits_{k} {\sum\nolimits_{l} {p_{k} p_{l} \left( {g_{i,l} - g_{k,l} } \right)} } ,$$

where ${p}_{k}$ and ${p}_{l}$ are the frequency of the alleles $k$ and $l$, respectively, and ${g}_{i,l}$ (${g}_{k,l}$) is the genotypic RSS of the genotype consisting of alleles $i$ and $l$ ($k$ and $l$). The breeding value of RSS for an individual is given as:

2 $$a_{j} \, = \,\sum\nolimits_{i} {\gamma_{i,j} \alpha_{i} } ,$$

where ${\gamma }_{i,j}$ is the number of copies of allele $i$ in animal $j$.

It follows from Eq. (1), that the estimated mean allele effects are:

3 $$\widehat{\alpha}_{i}\, = \,\sum\nolimits_{k} {\sum\nolimits_{l} {\hat{p}_{k} \hat{p}_{l} \left( {\hat{g}_{i,l} - \hat{g}_{k,l} } \right)} } ,$$

where ${\hat{p}}_{k}$, ${\hat{p}}_{l}$, ${\hat{g}}_{i,l}$, and ${\hat{g}}_{k,l}$ are estimates of ${p}_{k}$, ${p}_{l}$, ${g}_{i,l}$, and ${g}_{k,l}$, respectively. Having ${\hat{\alpha }}_{i}$, the estimated breeding values (EBV) for RSS of individual *j* are calculated as:

4 $$\hat{a}_{j} \, = \,\sum\nolimits_{i} {\hat{\gamma }_{i,j} \hat{\alpha }_{i} } ,$$

where the summation is over all possible alleles and ${\hat{\gamma }}_{i,j}$ is an estimate of ${\gamma }_{i,j}$.

Therefore, to compute EBV for RSS, we need ${\hat{\gamma }}_{i,j}$ for the animals, ${\hat{p}}_{i}$ for all *PRNP* alleles in the population, and ${\hat{g}}_{k,l}$ for every *PRNP* genotype in the population.

### Predicting number of copies of *PRNP* alleles

We used a linear model for predicting the allele content [12, 15] for $n-1$ out of $n$ alleles of *PRNP*:

5 $$\left( {\begin{array}{*{20}c} {{\mathbf{y}}_{1} } \\ \vdots \\ {{\mathbf{y}}_{n - 1} } \\ \end{array} } \right) = {\mathbf{X}}\left( {\begin{array}{*{20}c} {\mu_{1} } \\ \vdots \\ {\mu_{n - 1} } \\ \end{array} } \right) + {\mathbf{Z}}\left( {\begin{array}{*{20}c} {{\mathbf{u}}_{1} } \\ \vdots \\ {{\mathbf{u}}_{n - 1} } \\ \end{array} } \right) + \left( {\begin{array}{*{20}c} {{\mathbf{e}}_{1} } \\ \vdots \\ {{\mathbf{e}}_{n - 1} } \\ \end{array} } \right),$$

where ${\mathbf{y}}_{i}$ is a vector of allele content for allele $i$ for all genotyped individuals, $\mathbf{X}$ is an incidence matrix that links the genotypes to the means, $$\left(\begin{array}{c}{\mu }_{1}\\ \vdots \\ {\mu }_{n-1}\end{array}\right)$$ are the trait means (equal to $2{p}_{i}$, where ${p}_{i}$ is the allele frequency of the counted allele), $$\mathbf{Z}=\left(\begin{array}{ccc}{\mathbf{Z}}_{1}& \cdots & {\mathbf{0}}\\ \vdots & \ddots & \vdots \\ {\mathbf{0}}& \cdots & {\mathbf{Z}}_{n-1}\end{array}\right)$$ is an incidence matrix connecting the genotypes to individuals, $$\left(\begin{array}{c}{\mathbf{u}}_{1}\\ \vdots \\ {\mathbf{u}}_{n-1}\end{array}\right)$$ is a vector of allele content as deviation from $$\left(\begin{array}{c}{\mu }_{1}\\ \vdots \\ {\mu }_{n-1}\end{array}\right)$$, and $$\left(\begin{array}{c}{\mathbf{e}}_{1}\\ \vdots \\ {\mathbf{e}}_{n-1}\end{array}\right)$$ is a vector of random residuals. The assumed (co)variance structure for the breeding values was:

6 $$Cov\left( {\begin{array}{*{20}c} {{\mathbf{u}}_{1} } \\ \vdots \\ {{\mathbf{u}}_{n - 1} } \\ \end{array} } \right) = \left( {\begin{array}{*{20}c} {2p_{1} q_{1} } & \cdots & { - 2p_{1} p_{n - 1} } \\ \vdots & \ddots & \vdots \\ { - 2p_{1} p_{n - 1} } & \cdots & {2p_{n - 1} q_{n - 1} } \\ \end{array} } \right) \otimes {\mathbf{A}},$$

where $\otimes$ is the Kronecker product, $\mathbf{A}$ is the numerator relationship matrix and ${q}_{i}=1-{p}_{i}$. For the method to work using BLUP, the residual ${\mathbf{e}}_{i}$ cannot be equal to zero, despite the allele content theoretically having a heritability of 1 [12]. Therefore, we assume that ${h}^{2}<1$ and

7 $$Cov\left( {\begin{array}{*{20}c} {{\mathbf{e}}_{1} } \\ \vdots \\ {{\mathbf{e}}_{n - 1} } \\ \end{array} } \right) = \frac{{1 - h^{2} }}{{h^{2} }}\left( {\begin{array}{*{20}c} {2p_{1} q_{1} } & \cdots & 0 \\ \vdots & \ddots & \vdots \\ 0 & \cdots & {2p_{n - 1} q_{n - 1} } \\ \end{array} } \right) \otimes {\mathbf{I}},$$

where ${h}^{2}$ is the assumed heritability. We applied the model assuming three values for ${h}^{2}$, 0.90, 0.95 and 0.99. By solving this model, we get estimates of ${\mathbf{u}}_{i}$, i.e., ${\widehat{\mathbf{u}}}_{i}$**,** for $n-1$ alleles. Subsequently, we get the estimated allele content as ${\hat{{\varvec{\upgamma}}}}_{i}={\widehat{\mathbf{u}}}_{i}+{\mu }_{i}{\mathbf{1}}$ where ${\hat{{\varvec{\upgamma}}}}_{i}$ is a vector of predictions of allele content and ${\mathbf{1}}$ is a vector of ones. For the ${n}^{th}$ allele, we found the estimates using ${\hat{{\varvec{\upgamma}}}}_{n}=2-\sum_{i=1}^{n-1}{\hat{{\varvec{\upgamma}}}}_{i}$.

### Relative resistance to scrapie of *PRNP* genotypes

Our estimation of the RSS values associated with different genotypes (genotypic RSS) was based on published odds-ratio estimates of detected scrapie infection, using the wild type ARQ/ARQ genotype as reference. From that, we calculated the mean allele effects of the *PRNP* alleles using Eq. (3).

We compiled published estimates of genotypic RSS from 10 publications. To identify useful publications, we searched the PubMed database for the words “scrapie”, "sheep”, and “genotype”. After screening the titles of the 673 publications, and subsequently the abstracts and result sections of selected papers, we narrowed the selection down to the 10 publications presenting scrapie risk comparisons between different genotypes. When individual flock results were provided, we treated each flock as a unique estimate. For studies presenting collective results without individual flock data, we used the pooled estimates. We excluded any comparisons with less than 4 scrapie cases. From these studies, we extracted the number of healthy and affected sheep for the genotypes present in at least three sheep for the group. The studies are listed in Table 1. We conducted a meta-analysis, combining the estimated odds-ratios for each genotype compared to the homozygous wildtype (ARQ/ARQ) genotype. For the meta-analysis, we used the meta package [25] in the R [26] computing environment. We used the fixed effects model with the Mantel–Haenszel [27] method to calculate the common effect across studies.

**Table 1:** Overview of the papers used in meta-analysis

Paper	Country	Genotypes tested	Number of groups
Hunter et al. [19]	UK	ARR/ARR, ARR/AHQ, ARR/ARQ, ARR/VRQ, AHQ/AHQ, AHQ/ARQ, AHQ/VRQ, VRQ/ARQ, VRQ/VRQ	5
Billinis et al. [20]	Greece	ARR/ARQ, AHQ/ARQ, AHQ/VRQ, VRQ/VRQ	1
Hagenaars et al. [9]	The Netherlands	ARR/ARR, ARR/AHQ, ARR/ARQ, ARR/VRQ, AHQ/AHQ, AHQ/ARQ, AHQ/VRQ, VRQ/ARQ, VRQ/VRQ	1
Harrington et al. [21]	Canada	ARR/ARR, ARR/AHQ, ARR/ARQ, ARR/VRQ, AHQ/ARQ, VRQ/ARQ	1
Thorgeirsdottir et al. [4]	Iceland	AHQ/ARQ, VRQ/ARQ, VRQ/VRQ	1
Tranulis et al. [22]	Norway	ARR/ARR, ARR/AHQ, ARR/ARQ, ARR/VRQ, AHQ/ARQ, AHQ/VRQ, VRQ/ARQ, VRQ/VRQ	2
Belt et al. [23]	The Netherlands	ARR/ARR, ARR/ARQ, ARR/VRQ, VRQ/ARQ, VRQ/VRQ	1
Acín et al. [24]	Spain	ARR/ARR, ARR/ARQ, ARR/VRQ, VRQ/ARQ	1
Baylis et al. [2]	UK	ARR/ARR, ARR/AHQ, ARR/ARQ, ARR/VRQ, AHQ/AHQ, AHQ/ARQ, AHQ/VRQ, VRQ/ARQ, VRQ/VRQ	1
Vaccari et al. [6]	Italy	T137/ARQ	5

For calculating the mean allele effects for RSS, we used the natural logarithm of the estimated odds-ratio from the meta-analysis as the genotypic RSS. Among known *PRNP* genotypes in Icelandic sheep, no estimates existed for genotypes with the C151 and N138 alleles. Further, the only genotype with the T137 allele with reported epidemical data was T137/ARQ. In cases lacking data, we assumed conservative values for genotypic RSS, i.e. more susceptibility to scrapie than expected from extrapolation, in vitro results, or unpublished data. Thus, we assumed that T137/ARR, T137/T137, and T137/AHQ had the same genotypic RSS as T137/ARQ, and that T137/VRQ was equivalent to VRQ/ARQ. Previous studies have suggested that sheep carrying the C151 allele are at least partially resistant to scrapie [4, 8, 28]. Thus, we assumed that the C151 allele is equivalent to the AHQ allele. Previous studies [4, 8] have not shown a clear difference between risk of scrapie infection in sheep carrying the N138 allele relative to the wild type allele ARQ. Therefore, we assumed that N138 is equivalent to ARQ.

### Simulation

For testing the models, we simulated a population with known genotypes. To create a realistic population structure, we based the simulation on a part of the registered pedigree of the Icelandic sheep population. We identified the breeding rams and ewes born in 2021 that had registered parents and all four grandparents in the pedigree. From these animals, we traced the pedigree for up to 10 generations. This resulted in pedigree information on 870,971 sheep for the simulation. The simulations were conducted 10 times (always using the same pedigree) and the presented results are averages of the 10 replicates.

We simulated genotypes for a single locus with 5 alleles, denoted A1, A2, A3, A4, and A5. The founder animals of the pedigree (72,727 animals) received alleles at random with probabilities 0.15, 0.05, 0.01, 0.001 and 0.789 for A1, A2, A3, A4, and A5, respectively. Subsequently, the remaining animals received their genotype based on pedigree information using the gene drop method. The simulations were done using custom-made software written in Fortran.

We simulated seven scenarios, varying in assumed genotyping strategy, in the accuracy of the pedigree information, and in the rate of genotyping errors. The scenarios are summarized in Table 2. The rand, h20 and h40 scenarios all had no errors in pedigree records or genotyping but differed in the genotyping strategy. The rand scenario had randomly selected 5% of all animals genotyped, the h40 scenario had 40% of rams genotyped but no ewes, and the h20 scenario had 20% of rams genotyped but no ewes. Other scenarios had the same genotyping strategy as h40 but included errors; 5% errors in recorded pedigree (hr40x1), 10% errors in recorded pedigree (hr40x2), 1% genotyping errors (hr40y1), and 2% genotyping errors (hr40y2). For simulating the pedigree recording errors, two types of errors were introduced. First, we made 80% of the pedigree errors by swapping the registered sire for another ram which had offspring in the same flock in the same year. Second, we made 20% of the errors by swapping parents between animals within flock and year. We simulated the genotyping errors by replacing the true genotype with another genotype, with chances of the other genotypes being equal to their genotypic frequencies. In all scenarios, no animals born in 2021 (the last year included in the data) were assumed to be genotyped.

**Table 2:** Simulation scenarios tested

Scenario	Description	N. of genotyped*
Rand	5% of all animals genotyped at random	40,440
hr20	20% of rams genotyped at random	23,278
hr40	40% of rams genotyped at random	46,437
hr40x1	40% of rams genotyped at random, 5% pedigree errors	46,437
hr40x2	40% of rams genotyped at random, 10% pedigree errors	46,437
hr40y1	40% of rams genotyped at random, 1% genotyping errors	46,437
hr40y2	40% of rams genotyped at random, 2% genotyping errors	46,437

^*^Number of genotyped animals in each scenario, average over 10 replicates

For each scenario in the simulated data, we predicted the allele content for ungenotyped animals for alleles A1, A2, A3 and A4 using Eq. (5). We calculated the variance components from the simulated allele frequencies using Eqs. (6 and 7). We tested three different values for assumed heritability, 0.90, 0.95 and 0.99. To get the BLUP allele content predictions we used the dmu5 program of the DMU package [29]. The predictions were evaluated separately for two validation groups, namely sheep born in 2021, i.e. selection candidates younger than any genotyped animal (63,633 animals), and ungenotyped animals born in 2016–2020, i.e. ungenotyped animals contemporary to genotyped animals (approximately 290,000 individuals, exact number depended on scenario and varied between replicates). We tested the accuracy and dispersion of the prediction of allele content as correlation between true and predicted allele content and regression of true allele content on predicted, respectively.

We tested the ability of the methods to predict RSS breeding values by assigning genotypic effects of RSS to the simulated genotypes. We used the results from the meta-analysis described above. We assigned RSS values to the genotypes assuming that the A1 allele was AHQ, A2 was VRQ, A3 was T137, A4 was ARR and A5 was ARQ. We calculated the mean allele effects of the alleles using Eq. (3) with the estimated RSS of the genotypes and the allele frequencies used for the simulation. From these mean allele effects, we calculated the true breeding values for RSS for the simulated individuals using Eq. (2).

The RSS of the genotypes were estimated and were not known without error. Therefore, we tested three scenarios for predicting the breeding value for RSS. In one scenario, we assumed that the estimated genotypic RSS from the meta-analysis were both the true and the estimated genotypic RSS. We name this scenario trueRSS. In the other two scenarios we assumed that the estimated genotypic RSS from the meta-analysis were the true genotypic RSS effects, but the estimated RSS for calculating EBV in each replicate were sampled from normal distributions for each replicate. We name these scenarios estRSS1 and estRSS2. For both estRSS1 and estRSS2, we assumed that the true genotypic RSS was the mean of the distribution for sampling the effects. For estRSS1, the standard error of the RSS estimates (on the logarithmic scale) was the standard deviation of the distribution. For the genotypes involving the A3 allele (set as T137), where the RSS estimate was not based on data but assumed to be the same as other genotypes, we set the standard error as 1.0, which was larger than the standard error for any of the RSS estimates. For the estRSS2 scenario, the standard deviation of the distribution was set to 1.5 for all genotypes.

For the trueRSS, estRSS1, and estRSS2 scenarios, we tested the results of the seven scenarios for predicting allele content. We tested the accuracy and dispersion of the predictions as the correlation between true breeding values and the EBV and the regression coefficient of true breeding value on the EBV, respectively.

### Testing on empirical data

Genotypes of *PRNP* for 47,782 sheep born in the years 1987 until 2023 were available for this study. Of these, around 7000 were genotyped only for the polymorphisms at codons 136 and 154 (i.e., distinguishing VRQ and AHQ, respectively, from ARQ), so the allele content for the other alleles was missing. We had access to pedigree records from the database of the Farmers Association of Iceland with pedigree records for 2.6 million ewes and breeding rams born in 1980 to 2022. We pruned the pedigree, only including genotyped sheep and their ancestors 10 generations back. After pruning, the pedigree file consisted of information on 221,569 sheep.

In an attempt to assess the accuracy of the genotyping and recorded pedigree, we estimated the heritability of the allele content for a single allele at a time using a single-trait model. The single trait model for allele $i$ was

$${\mathbf{y}}_{i} = {\mathbf{1}}\mu_{i} + {\mathbf{Z}}_{i} {\mathbf{u}}_{i} + {\mathbf{e}}_{i}$$

where the terms are as described for Eq. (5). We used AI-REML with the DMUAI procedure of the DMU package [29] for estimating variance components.

For validating the predictions of allele content, we masked the genotypes of three alternative validation populations. The first validation scenario tested predictions of animals contemporary to genotyped animals. Then, the validation population consisted of females born in 2021 and 2022, genotyped with methods that detect all alleles of *PRNP* known in Iceland, in total 8068 sheep. The training population consisted of 32,269 sheep born in 2020 and earlier as well as rams born in 2021 and 2022. The second validation population consisted of sheep born in 2022 genotyped with methods that detected all alleles of *PRNP* known in Iceland, in total 4816 sheep. The training population consisted of 35,798 sheep born in 2021 and earlier. The third validation population consisted of sheep born in 2023 genotyped with methods that detected all alleles of *PRNP* known in Iceland, in total 6677 sheep. The training population consisted of 40,623 sheep born in 2022 and earlier. At the time of data extraction, the available genotype data for sheep born in 2023 was predominantly from strategic genotyping of offspring from planned mating of sheep heterozygous for rare alleles, predominantly ARR and T137.

The variance components for the validation prediction were based on allele frequencies and Eqs. (6) and (7) with varying assumed heritability of 0.99, 0.95 and 0.90. Obtaining unbiased estimates of allele frequencies from the database was challenging because of strategic genotyping of offspring of heterozygous carriers of rare alleles. Therefore, we based the input allele frequencies to calculate the variance components on the genotype frequencies from a population wide genotyping initiative presented by Einarsson [5]. We used the dmu5 program from the DMU package [29] to solve the equations.

We tested the accuracy and dispersion of the prediction of allele content as correlation between the allele content based on the genotyping and the predicted allele content, and the regression coefficient of the allele content based on genotyping on predicted allele content, respectively. We calculated standard deviations for the correlations and regression coefficients from 10,000 bootstrap samples.

For predicting RSS EBV for the real data, we assumed that the RSS estimates for the genotypes were the true values. The allele frequency for calculating $\widehat{{\alpha }_{i}}$ using Eq. (3) should be from the current population and be unbiased. To avoid bias because of selective genotyping while having an estimate of the current allele frequencies, we used the predicted allele content in the last year of the training population ${\widehat{p}}_{i}=0.5\times \overline{{\widehat{\upgamma } }_{i}}$, where $\overline{{\widehat{\upgamma } }_{i}}$ is the average of ${\hat{{\varvec{\upgamma}}}}_{i}$.

## Results

### Relative susceptibility of *PRNP* genotypes

Table 3 presents the estimates of the natural logarithm of the odds-ratio of scrapie for various genotypes compared with the ARQ/ARQ genotype, from the meta-analysis. The homozygous ARR genotype had the lowest RSS, while the homozygous VRQ genotype had the highest RSS. With the AHQ/VRQ genotype as one exception, the heterozygous genotypes had estimated RSS values that fell between those of the homozygous genotypes.

**Table 3:** Estimated relative scrapie susceptibility of different genotypes from meta-analysis of results from published papers

Genotype	RSS(SE)*	Number of comparisons
ARR/ARR	−4.74 (0.53)	11
ARR/AHQ	−3.65 (0.56)	6
ARR/ARQ	−3.53 (0.23)	12
ARR/VRQ	−0.26 (0.14)	10
AHQ/AHQ	−1.94 (0.40)	3
AHQ/ARQ	−1.48 (0.16)	10
AHQ/VRQ	−2.41 (0.35)	5
VRQ/ARQ	1.81 (0.07)	12
VRQ/VRQ	2.45 (0.11)	8
T137/VRQ	−3.01 (0.64)	5

^*^ Natural logarithm of the odds ratio indicating relative scrapie susceptibility compared with ARQ/ARQ genotype

### Validation results from simulated data

**Fig. 1:**
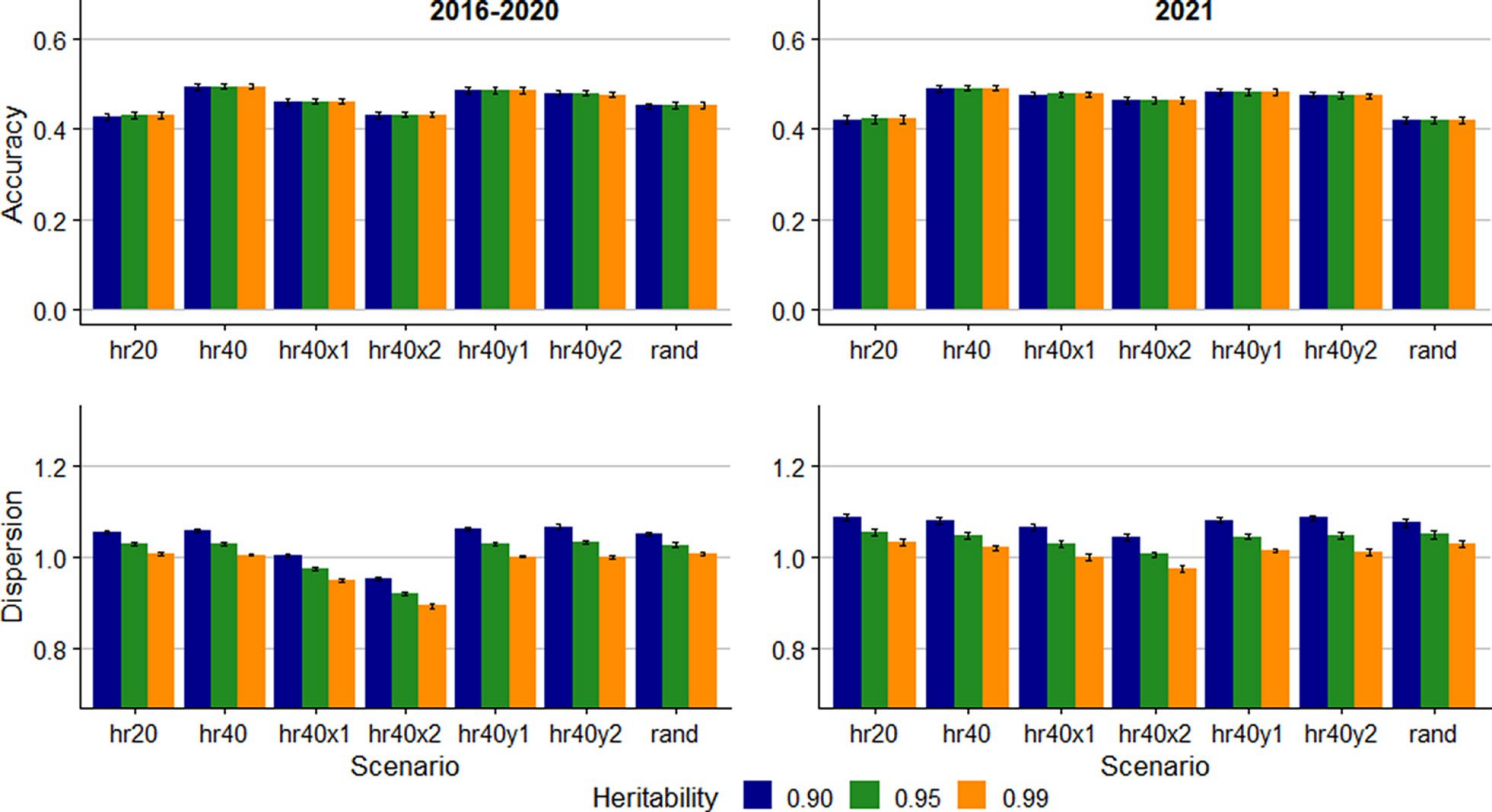
Accuracy and dispersion bias for predicting the number copies of the A1 allele. Error bars represent standard errors across 10 replicates. hr20: 20% of rams genotyped; hr40: 40% of rams genotyped; hr40x1: 40% of rams genotyped and 5% incorrect pedigree; hr40x2: 40% of rams genotyped and 10% incorrect pedigree; hr40y1: 40% of rams genotyped and 1% incorrect genotype; hr40y2: 40% of rams genotyped and 2% incorrect genotype; rand: 5% breeding stock (male and female) genotyped

Figure 1 shows the accuracy and dispersion for predictions of allele content of the A1 allele. The accuracy was highest in the hr40 scenario, 0.49 for both the 2021 and 2016–2020 groups. Lower genotyping proportion, errors in recorded pedigree, genotyping errors, and random genotyping of ewes and rams all resulted in lower accuracy. For the 2016–2021 group, reduced genotyping (hr20) and 10% pedigree errors (hr40x2) resulted in the lowest accuracy, 0.43. For the 2021 group, pedigree errors had less impact on the accuracy than in the 2016–2020 group. The prediction accuracy for A1 was not affected by the assumed heritability. However, the dispersion was affected by the heritability assumptions, as lower assumed heritability resulted in higher regression coefficient for all scenarios. For the scenarios where the pedigree data were accurate, assuming a heritability of 0.99 resulted in regression coefficients nearing 1.0.

**Fig. 2:**
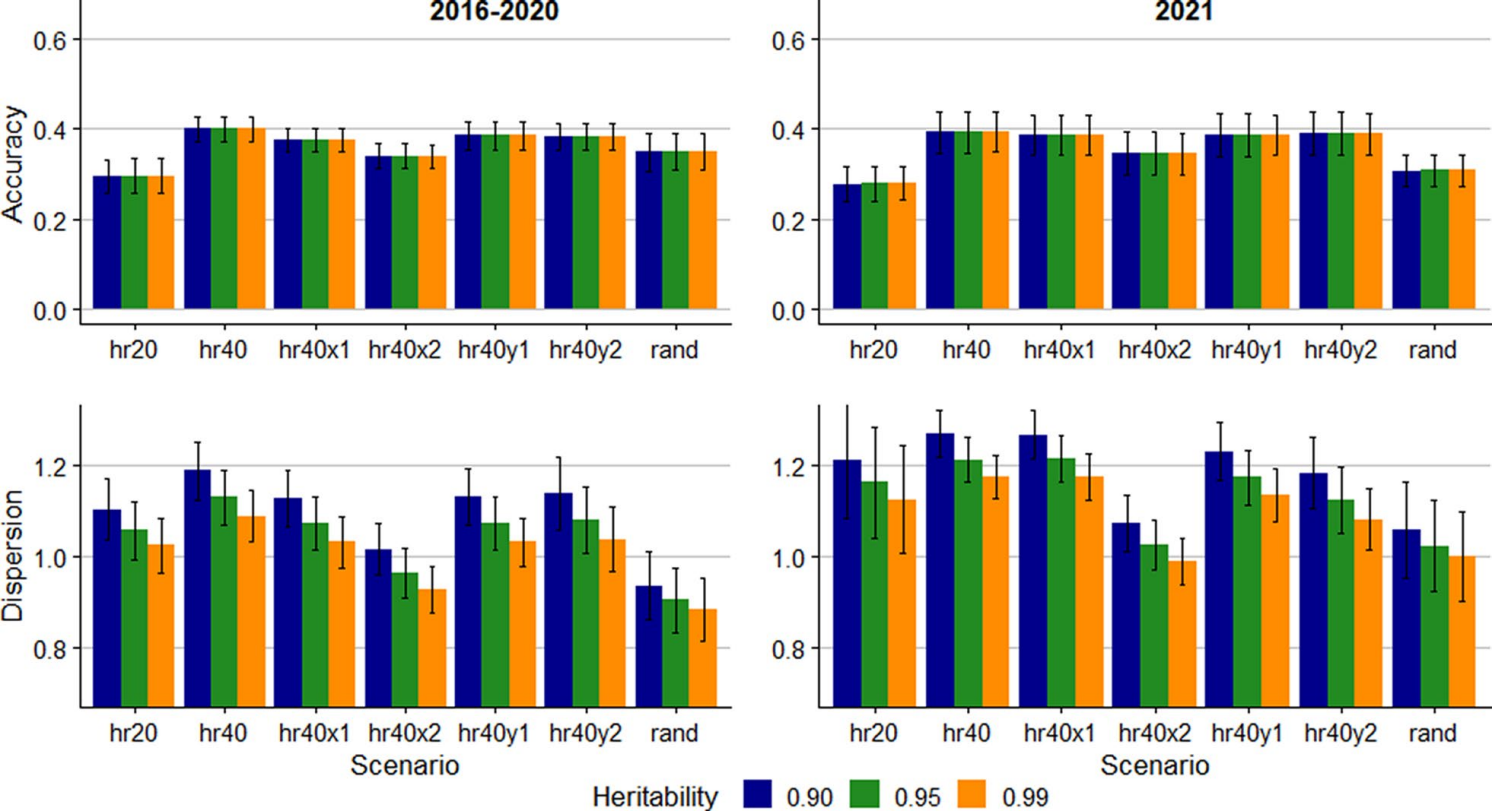
Accuracy and dispersion bias for predicting the number of copies of the A4 allele (very rare) in ungenotyped sheep. Error bars represent standard errors across 10 replicates. hr20: 20% of rams genotyped; hr40: 40% of rams genotyped; hr40x1: 40% of rams genotyped and 5% incorrect pedigree; hr40x2: 40% of rams genotyped and 10% incorrect pedigree; hr40y1: 40% of rams genotyped and 1% incorrect genotype; hr40y2: 40% of rams genotyped and 2% incorrect genotype; rand: 5% breeding stock (male and female) genotyped

Figure 2 shows the accuracy and dispersion of prediction of the allele content of the rare A4 allele. The results for the A4 allele fluctuated notably across replicates, as reflected by the large standard error bars in Fig. 2. Accuracies for the A4 allele were universally lower than for the A1 allele across all scenarios and both groups, ranging from 0.28 to 0.40. Additionally, the regression coefficients which indicate dispersion deviated more from 1.0 for A4 than for A1, but they followed the same trend with lower coefficients following higher assumed heritability.

The prediction accuracies and dispersion for the A2 allele are available in Additional file 1, Figure S1. The accuracies for A2 were similar or slightly lower than that for the A1 allele, while the regression coefficients where notably lower for A2, particularly in the hr40y1 and hr40y2 scenarios. The prediction accuracies and dispersion for the A3 allele are available in Additional file 2, Figure S2. The accuracies for the A3 allele fall in most instances between the numbers for the A1 and A4 alleles. The prediction accuracies and dispersion for A5, the most common allele, are available in Additional file 3, Figure S3. The accuracy and dispersion results for the A5 allele are similar to those for A1.

**Fig. 3:**
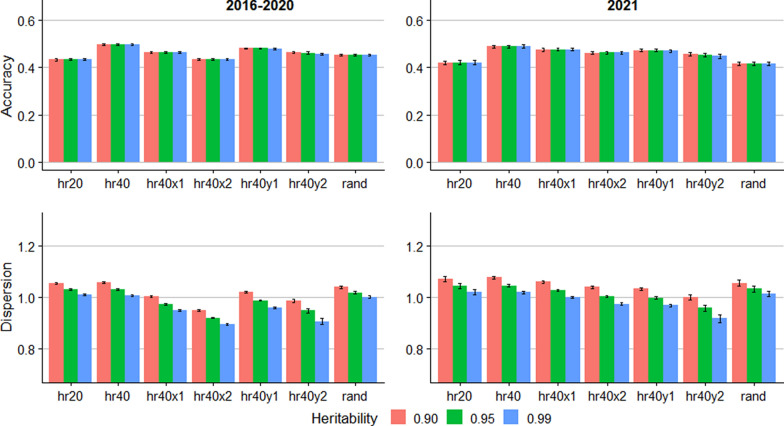
Accuracy and dispersion bias for predicting the breeding value of susceptibility for scrapie in ungenotyped sheep, assuming that the relative susceptibility of the *PRNP* genotypes is known without error. Error bars represent standard errors across 10 replicates. hr20: 20% of rams genotyped; hr40: 40% of rams genotyped; hr40x1: 40% of rams genotyped and 5% incorrect pedigree; hr40x2: 40% of rams genotyped and 10% incorrect pedigree; hr40y1: 40% of rams genotyped and 1% incorrect genotype; hr40y2: 40% of rams genotyped and 2% incorrect genotype; rand: 5% breeding stock (male and female) genotyped

The prediction accuracy and dispersion of RSS EBV from the trueRSS scenarios are presented in Fig. 3. As for the allele content, the assumed heritability did not impact the accuracy. For the 2016–2020 group, the accuracy was lowest for the hr20 scenario (0.43), while the accuracy was highest for the hr40 scenario (0.49–0.50). For the 2021 group, the accuracy was lowest for the rand scenario (0.41) and the hr20 scenario (0.42). For the 2016 to 2020 group, the regression coefficients indicating dispersion were close to 1.0 for the scenarios without errors in pedigree or genotype when the assumed heritability was 0.99. Assuming lower heritability generated deflated EBV for this group. In other scenarios, lower assumed heritability resulted in regression coefficients closer to 1.0. For the 2021 group, however, assuming heritability of 0.99 resulted in regression coefficient closest to 1.0 for hr40x1, whereas assumed heritability of 0.95 resulted in the least dispersion for the hr40x2 scenario.

**Fig. 4:**
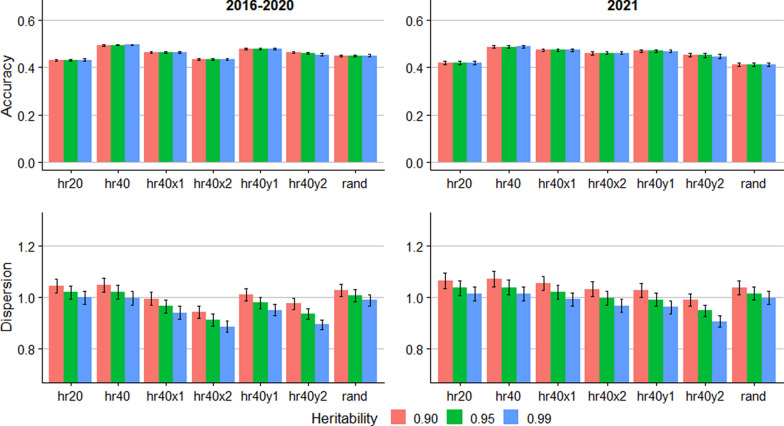
Accuracy and dispersion bias for predicting the breeding value of susceptibility for scrapie in ungenotyped sheep in the estRSS1 scenario, assuming small error in estimated genotypic relative scrapie susceptibility. Error bars represent standard errors across 10 replicates. hr20: 20% of rams genotyped; hr40: 40% of rams genotyped; hr40x1: 40% of rams genotyped and 5% incorrect pedigree; hr40x2: 40% of rams genotyped and 10% incorrect pedigree; hr40y1: 40% of rams genotyped and 1% incorrect genotype; hr40y2: 40% of rams genotyped and 2% incorrect genotype; rand: 5% breeding stock (male and female) genotyped

Figure 4 shows the accuracy and dispersion for RSS EBV in the estRSS1 scenarios. The prediction accuracy was only minimally lower compared to trueRSS for both groups and all scenarios. The accuracy of RSS EBV was 0.49 in the hr40 scenario. Reducing the number of genotyped rams by half, or genotyping rams and ewes randomly, both reduced the accuracy, with accuracies of 0.42 and 0.41 in the hr20 and rand scenarios, respectively. Similarly, pedigree errors and genotyping errors decreased accuracy with accuracies of 0.46 and 0.45 in the hr40x2 and hr40y2 scenarios, respectively. For the dispersion, the standard error across the 10 replicates was considerably higher in estRSS1 compared to trueRSS, indicating lack of stability.

**Fig. 5:**
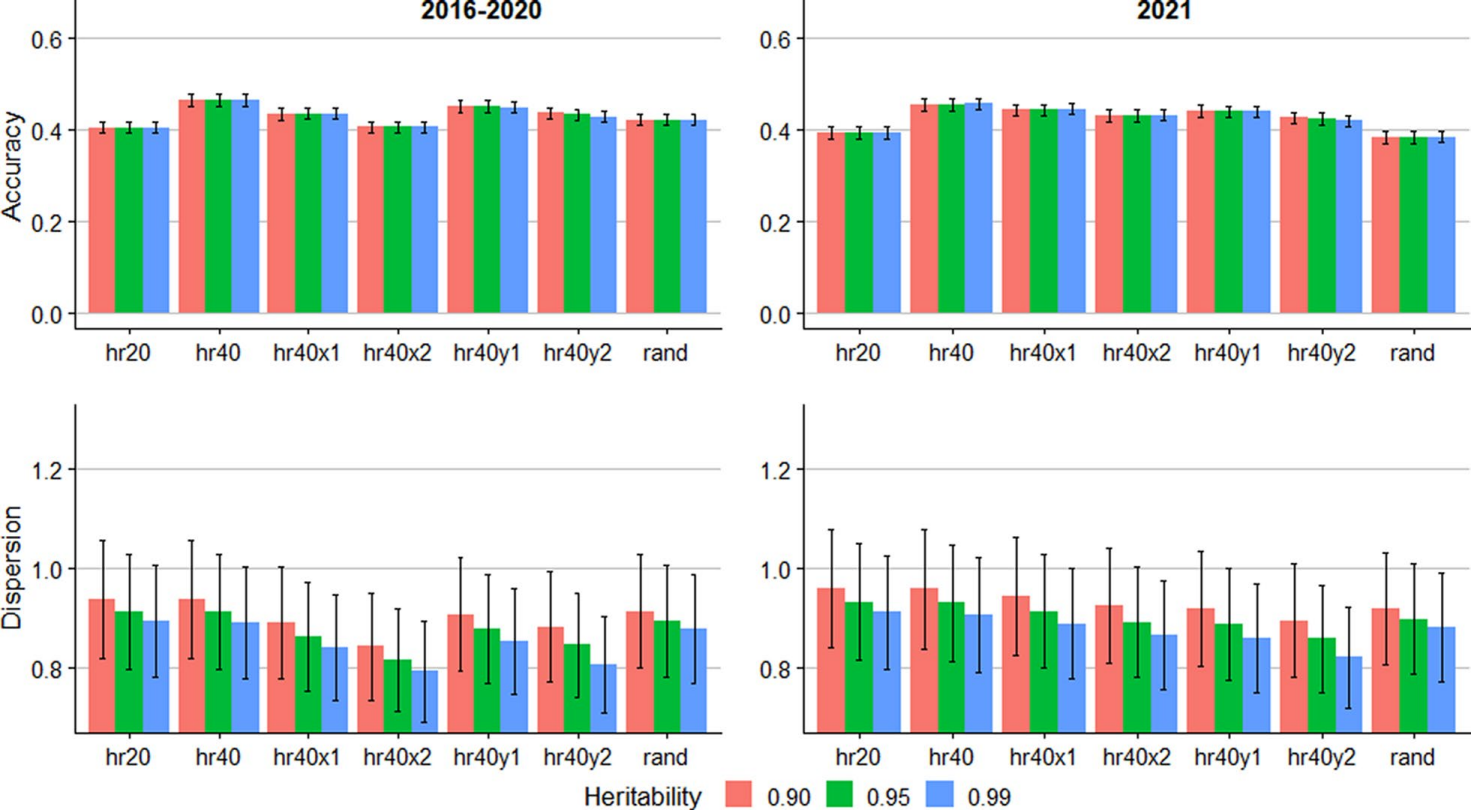
Accuracy and dispersion bias for predicting the breeding value of susceptibility for scrapie in ungenotyped sheep in the estRSS2 scenario, assuming large error in estimated genotypic relative scrapie susceptibility. Error bars represent standard errors across 10 replicates. hr20: 20% of rams genotyped; hr40: 40% of rams genotyped; hr40x1: 40% of rams genotyped and 5% incorrect pedigree; hr40x2: 40% of rams genotyped and 10% incorrect pedigree; hr40y1: 40% of rams genotyped and 1% incorrect genotype; hr40y2: 40% of rams genotyped and 2% incorrect genotype; rand: 5% breeding stock (male and female) genotyped

Figure 5 shows the accuracy and dispersion for RSS EBV in the estRSS2 scenarios. The accuracies were lower than from the trueRSS and estRSS1 scenarios, lowest in the 2021 group in the hr20 and rand scenarios (0.39). The EBV were inflated for all scenarios, with the regression coefficients in the range 0.79 (hr40x2 in the 2016–2020 group assuming heritability of 0.99) to 0.96 (hr40 in the 2021 validation group assuming heritability of 0.90).

### Results from empirical data

Table 4 shows the estimated heritability of allele content for the *PRNP* alleles. The heritability estimates for the three rarest alleles were relatively low: 0.17 for ARR, 0.46 for T137 and 0.73 for C151. Conversely, the estimates were higher for the more common alleles, highest for VRQ, (1.00).

**Table 4:** Estimated genetic variance and heritability for allele content for *PRNP,* allele frequency and mean allele effect for sheep born in 2021

Alleles	Genetic variance	Heritability	Allele frequency*	Mean allele effect*
VRQ	0.089	1.00	0.035	1.62
T137	0.005	0.46	0.001	−2.71
N138	0.103	0.93	0.054	0.07
C151	0.009	0.73	0.005	−1.42
AHQ	0.117	0.87	0.078	−1.42
ARR	0.004	0.17	0.0001	−3.29
ARQ			0.827	0.07

^*^Mean allele effect for sheep born in 2021. Allele frequencies were calculated from predictions of the linear model for genotyped and ungenotyped sheep born that year

**Fig. 6:**
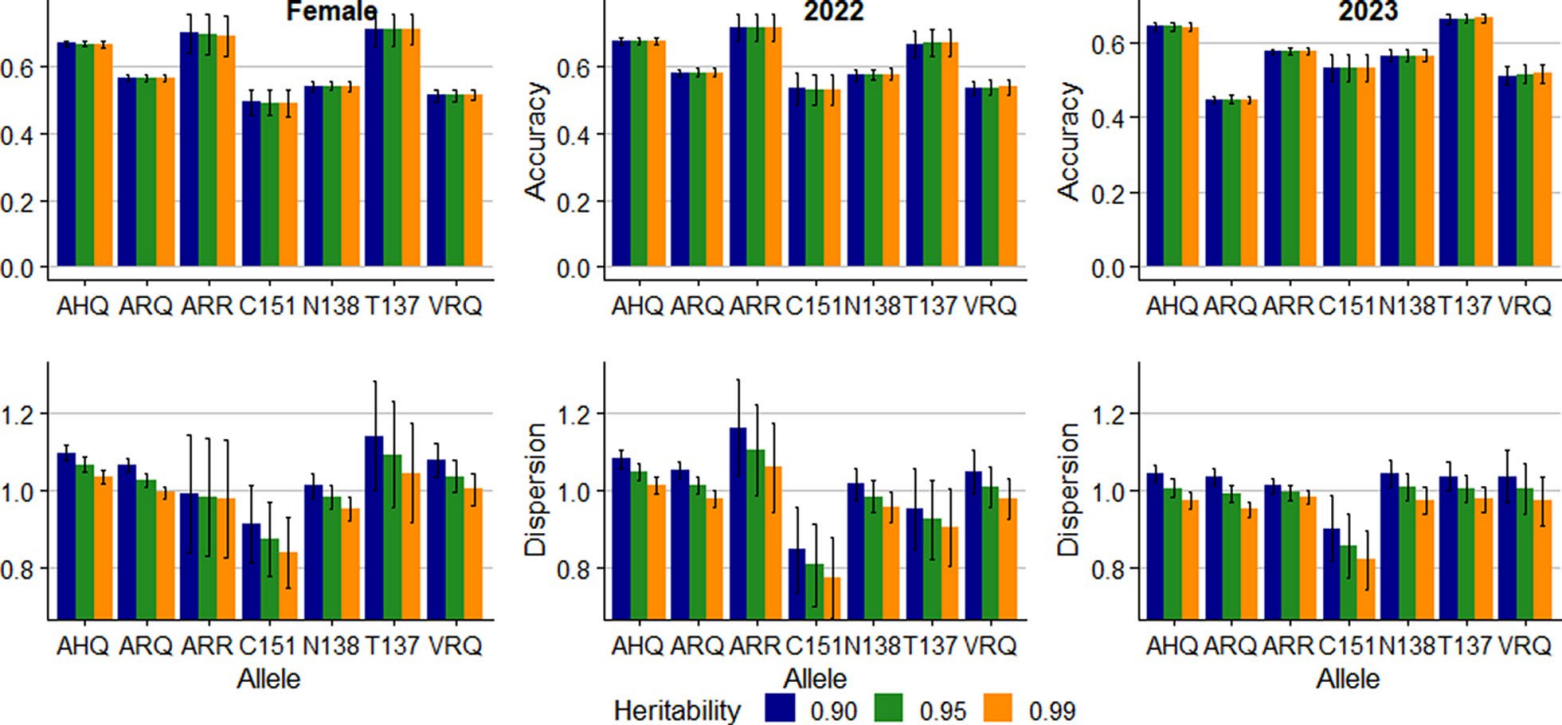
Accuracy and dispersion bias of prediction of number of copies of *PRNP* alleles in Icelandic sheep. Error bars show standard deviations from 10,000 bootstrap samples

Figure 6 shows the prediction accuracy and dispersion for allele content for empirical data. While the assumed heritability did not affect the accuracy, a lower assumed heritability resulted in larger regression coefficient for all alleles. The allele content for the T137, ARR and AHQ alleles was predicted with the highest accuracy for the female and 2022 validation groups, with accuracy exceeding 0.65. However, standard deviations of accuracy and dispersion estimates derived from 10,000 bootstrap samples were substantial for the rare alleles, i.e. ARR, C151 and T137. For the 2023 group, the accuracy was highest for the AHQ and T137 alleles, 0.64 and 0.66, respectively.

Table 4 shows the allele frequencies and mean allele effects for the *PRNP* alleles, based on the allele frequencies of sheep born in 2021. The alleles with the lowest mean RSS effect were very rare, allele frequency was 0.001 and 0.0001 for the T137 and ARR alleles, respectively.

Table 5 shows the accuracy and dispersion for the RSS EBV. The accuracy for the 2022 validation group was 0.65 irrespective of the assumed heritability. For the female validation group, the accuracy ranged from 0.63 to 0.64 across assumed heritability levels. However, for the 2023 group, the accuracy was lower, 0.46. The dispersion results suggested limited bias, the regression coefficients across assumed heritability and the two validation groups ranged from 0.97 to 1.09. The coefficient was closest to 1.00 when the assumed heritability was 0.99 for the female and 2022 groups but for the 2023 the coefficients were closer when assuming a heritability of 0.90 or 0.95.

**Table 5:** Accuracy and dispersion bias of estimated breeding values for relative scrapie susceptibility (standard deviations from 10,000 bootstrap samples)

Assumed heritability	Female 2021 to 2022		2022		2023	
	Accuracy	Dispersion	Accuracy	Dispersion	Accuracy	Dispersion
0.90	0.64 (0.01)	1.09 (0.02)	0.65 (0.01)	1.06 (0.02)	0.46 (0.01)	1.01 (0.02)
0.95	0.64 (0.01)	1.06 (0.02)	0.65 (0.01)	1.03 (0.02)	0.46 (0.01)	0.99 (0.02)
0.99	0.63 (0.01)	1.03 (0.02)	0.65 (0.01)	1.00 (0.02)	0.46 (0.01)	0.97 (0.02)

## Discussion

We have presented a procedure for calculating EBV for scrapie resistance in sheep, both genotyped and ungenotyped for *PRNP*. Our approach makes use of estimates of genotypic RSS from multiple studies, combining available data for effects that are generally not accurately estimated from single studies. Further, our method provides a single estimate of RSS EBV per animal, both genotyped and ungenotyped, facilitating straightforward implementation for breeding decisions.

### Predicting allele content

In contrast with the single-trait model in Boareki et al. [14], we used multi-trait models for predicting allele content. The multi-trait model could improve accuracy when some animals are only partially genotyped, i.e. not all known polymorphisms in the gene are checked. This was evident in our real data, where the older genotyping only checked for the common polymorphisms known to impact scrapie susceptibility.

The prediction accuracy was lower for the rare A4 allele compared with the other alleles in the simulated data (Fig. 2) and the accuracy in general decreased slightly with lower allele frequency. Lower accuracy with lower allele frequency aligns with the findings of Gengler et al. [12] and Boareki et al. [14]. The accuracy was more dependent on allele frequency in Boareki et al. [14], where accuracy of predicting the more common alleles was around 0.70, while for the rarest allele the accuracy was down to 0.41. However, the frequency of the rarest allele in their study [14] (0.05) was higher than for the A3 allele (${p}_{i}=0.01$) and the A4 allele (${p}_{i}=0.001$) in our study. Further, the population structure present in the pedigree that the simulations were based on could have affected the relationship between allele frequency and accuracy.

For the real data, the prediction accuracy for the allele content for the rare ARR and T137 alleles was among the highest (Fig. 6), albeit with large standard deviations across the bootstrap samples. The relatively high accuracy for these alleles, despite very low frequency, could be explained by the high proportion of relatives of carriers being genotyped. These alleles are rare and associated with low susceptibility for scrapie (Table 4), making breeders very keen on knowing all carriers of these alleles. Consequently, carriers and potential carriers in the validation population are likely to have genotyped close relatives in the training population.

For both empirical and simulated data, the dispersion of predicted allele content for rare alleles had large standard deviation and, in some cases, deviated considerably from 1.0. Consequently, in general, the linear model is more appropriate for alleles with moderate frequency than for rare alleles. Additionally, since potential carriers of rare alleles are few, anticipated cost reduction from selecting based on EBV rather than genotypes is limited. Therefore, using the linear model for prediction is of limited value in the case of rare alleles.

Assumptions about heritability did not affect the prediction accuracy, regardless of scenario and whether using simulated or real data. However, higher assumed heritability consistently lowered the regression coefficient indicating dispersion. Which assumed heritability resulted in regression coefficients closest to 1.00 varied across scenarios, and between alleles within scenarios. Therefore, these results do not permit drawing conclusions about the most suitable assumed heritability. The estimated heritability of allele content did not prove useful to use in the prediction models since the heritability estimates were highly variable across alleles (Table 4). The low heritability estimate for the ARR allele might be explained by the violation of assumptions of normality, which could have a more substantial impact for the rare allele than those with more moderate allele frequency. Further, because the allele is very rare, individual errors in pedigree or genotyping can have a major effect on the estimate. The estimates for the more common alleles were more reasonable. Still, the heritability estimates do not look convincing as a general quality control statistic for this data, as Forneris et al. [16] proposed for SNP data.

The proportion of genotyped sheep and the selection of which sheep were genotyped impacted the predictions. The accuracy of allele content predictions consistently proved lower for the hr20 scenario, where around 2.5% of the population was genotyped, compared to the hr40 scenario with approximately 5% genotyped. The proportion genotyped was higher for the empirical data, with 15–17% of the sheep in the pedigree genotyped, explaining the higher accuracy compared to the simulation. Boareki et al. [14] only assumed around 1.2% of the animals genotyped and still got reasonable accuracies, ranging from 0.4 to 0.7. Gengler et al. [12] found accuracies of 0.50 and 0.47 for allele frequencies of 0.4 and 0.2, respectively, with 12% of the animals genotyped. Discrepancies between studies are likely because of the effect of allele frequencies, genotyping strategy, population structure, or the validation group. The lower accuracies for the rand scenario compared with hr40, despite similar number of genotyped sheep, demonstrated the impact of genotyping strategy. Genotyping breeding rams is more effective because rams yield more offspring per breeding animal than ewes. Selection of rams was the main method in successful breeding programs for scrapie resistance [9, 10]. The validation groups differ between the studies. The 2021 group in the simulation study and the 2022 and 2023 groups in the empirical study were forward predictions of potential selection candidates, adhering to the standard for validation of genetic and genomic predictions [30, 31]. In contrast, Boareki et al. [14] and Gengler et al. [12] calculated the accuracy of all ungenotyped animals, irrespective of relevance for selection.

### Predicting breeding value for relative scrapie susceptibility

The mean allele effects (Table 4) of this study align somewhat with the additive effects of alleles assumed by Boareki et al. [14] for alleles common for both studies, despite differences in methodology. Still, our approach was based directly on data as far as possible. Furthermore, our predictions were defined as EBV and the mean allele effects accounts for the current allele frequency of the population.

For the T137/ARQ genotype, our RSS estimate was solely based on data from a single study in one breed in Italy [6]. This raises the possibility that the genotypic RSS associated with T137/ARR might be breed or scrapie-strain specific. Nonetheless, scrapie has not been detected in carriers of the allele in Iceland, suggesting that the allele is also associated with resistance in Iceland. For other genotypes including T137 we lacked useful data. It is worth noting that genotypes containing two rare alleles minimally impact estimates of mean allele effects. For genotypes involving C151 and N138 we had less data to use. Future research might provide more comprehensive data, enabling more precise estimates of the genotypic RSS for these genotypes.

Importantly, we predicted the breeding value for RSS, not the genotypic RSS. For selective breeding, the EBV is more relevant than the genotypic value [18]. However, the genotypic value might be more relevant for evaluating the flock resistance against scrapie outbreaks [17, 32].

The prediction accuracy for RSS EBV for trueRSS, estRSS1, and estRSS2 in the simulated data (Figs. 3, 4, and 5) ranged from 0.39 to 0.50 for all simulated scenarios. The limited range of the accuracies indicates that the methods retain moderate accuracy even with up to 10% pedigree errors, up to 2% genotyping errors, or without precise estimates of the genotypic RSS. Furthermore, in real data, which likely contains some pedigree and genotyping errors, the accuracy of RSS EBV was higher than for the simulations for two out of three validation groups (Table 5). However, the genotypic RSS was assumed to be known in the real data, but still the accuracy was higher than the simulated trueRSS scenario. Boareki et al. [14] reported an accuracy of 0.60 and 0.54 for scrapie resistance prediction, depending on the level of resistance in the simulated population, which is within the range of our results for empirical data but higher than the simulation results.

### Scrapie eradication

The focus of most scrapie eradication programs has been the ARR allele as the source of scrapie resistance [10]. The results of our meta-analysis support this strategy, the three genotypes associated with the lowest RSS were ARR/ARR, ARR/AHQ and ARR/ARQ (Table 3). However, ARR is rare or not present in some populations [3, 33]. For such populations, increasing the frequency of partially resistant genotypes could be useful for reducing the risk of scrapie. Nonetheless, whether complete prevention of scrapie outbreaks is possible with partially resistant genotypes is unknown. Findings from Hagenaars et al. [9, 34] suggest eradication is possible without universally resistant genotypes. Further, Hulst et al. [35] and Bijma et al. [36] showed that genetic selection for lower prevalence of infectious diseases with quantitative genetic effects is more effective than heritability estimates for susceptibility indicate. This is because of indirect genetic effects; reduced risk of an animal catching infectious disease reduces the risk that the animal spreads the disease in addition to reducing the risk of the animal itself to catch the disease.

A central assumption for determining RSS associated with genotypes is that scrapie strains do not affect the RSS associated with each genotype. However, different strains of scrapie exist and research results suggest their effect may be genotype dependent [32, 37, 38]. Nonetheless, results from different countries largely agree on the genotype ranking for RSS [2, 4, 9]. For the rarer alleles, RSS estimates either do not exist or were based on data from only one country which may reflect strain-specific RSS. Ideally, RSS estimates should originate from the same population where the RSS EBV are applied. However, single-population data is usually too limited for accurate genotypic RSS estimation.

The proposed RSS EBV can be presented the same way as EBV for production traits and thus potentially included in selection indices in addition to production traits. That would especially be relevant if unfavourable genetic correlations between RSS and production traits are present. The review of Sweeny and Hanrahan [39] did not find reports of strong correlations between *PRNP* genotypes and production traits, however, existence of such correlations has not been studied in the Icelandic populations.

Despite promising results of methods for predicting RSS EBV of ungenotyped animals, selection based on ram genotyping remains paramount for genetic selection for scrapie resistance [10]. Our results indicate that predictions from the linear model may be less accurate for rare alleles. Thus, genotyping resources should primarily target rams and potential carriers of rare alleles. For selection of replacement ewes and identification of potential carriers of resistant genotypes among ewes, the EBV could be valuable. Comparing the accuracies for the hr40 and rand scenarios (Figs. 3, 4, and 5) shows that accuracy is enhanced by genotyping rams rather than ewes and rams. Therefore, selecting rams based on genotyping and females based on EBV complement each other well.

## Conclusions

A linear model for predicting allele content in the *PRNP* gene, combined with estimates of relative susceptibility associated with *PRNP* genotypes can provide EBV for scrapie susceptibility for ungenotyped selection candidates with an accuracy of up to 0.65. This accuracy remains moderate, despite inaccurate pedigree, genotyping errors, or inaccurate genotypic RSS estimates. Providing RSS EBV can complement *PRNP* genotyping for gaining control of scrapie with genetic selection.

## Supplementary Information

Additional file 1. Accuracy and dispersion bias for predicting the number of copies of the A2 allele in ungenotyped sheep. Error bars represent standard errors across 10 replicates. hr20: 20% of rams genotyped; hr40: 40% of rams genotyped; hr40x1: 40% of rams genotyped and 5% incorrect pedigree; hr40x2: 40% of rams genotyped and 10% incorrect pedigree; hr40y1: 40% of rams genotyped and 1% incorrect genotype; hr40y2: 40% of rams genotyped and 2% incorrect genotype; rand: 5% breeding stock (male and female) genotyped.

Additional file 2. Accuracy and dispersion bias for predicting the number of copies of the A3 allele in ungenotyped sheep. Error bars represent standard errors across 10 replicates. hr20: 20% of rams genotyped; hr40: 40% of rams genotyped; hr40x1: 40% of rams genotyped and 5% incorrect pedigree; hr40x2: 40% of rams genotyped and 10% incorrect pedigree; hr40y1: 40% of rams genotyped and 1% incorrect genotype; hr40y2: 40% of rams genotyped and 2% incorrect genotype; rand: 5% breeding stock (male and female) genotyped.

Additional file 3. Accuracy and dispersion bias for predicting the number of copies of the A5 allele in ungenotyped sheep. Error bars represent standard errors across 10 replicates. hr20: 20% of rams genotyped; hr40: 40% of rams genotyped; hr40x1: 40% of rams genotyped and 5% incorrect pedigree; hr40x2: 40% of rams genotyped and 10% incorrect pedigree; hr40y1: 40% of rams genotyped and 1% incorrect genotype; hr40y2: 40% of rams genotyped and 2% incorrect genotype; rand: 5% breeding stock (male and female) genotyped.

## Data Availability

The empirical data is owned by the Icelandic Agricultural Advisory Centre and the Farmers Association of Iceland. Codes used for the simulations are available at https://github.com/jonhjalti/Scrapie_EBV.
