# Supplementary figures and images for: Predicted breeding values for relative scrapie susceptibility for genotyped and ungenotyped sheep
Source: Genet Sel Evol. 2024 Dec 18;56:77. doi: 10.1186/s12711-024-00947-x (PMC11656908; doi:10.1186/s12711-024-00947-x)

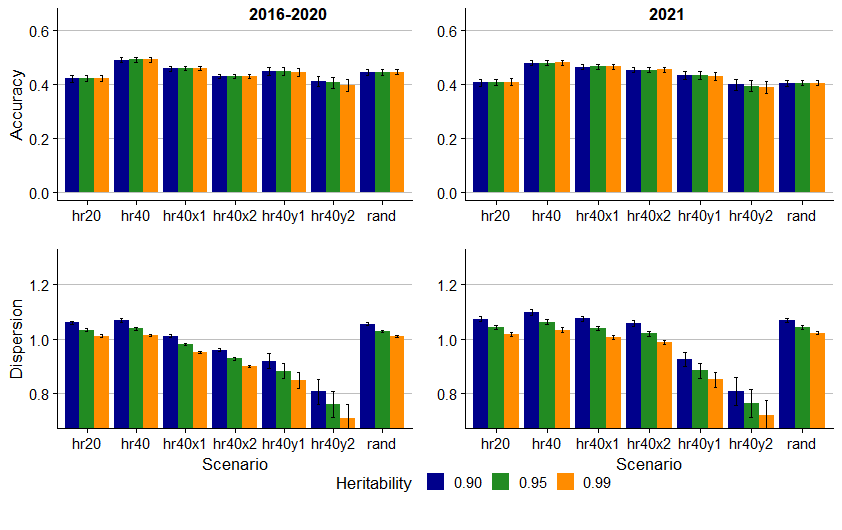

Supplement: Supplementary file 1 — Additional file 1. Accuracy and dispersion bias for predicting the number of copies of the A2 allele in ungenotyped sheep. Error bars represent standard errors across 10 replicates. hr20: 20% of rams genotyped; hr40: 40% of rams genotyped; hr40x1: 40% of rams genotyped and 5% incorrect pedigree; hr40x2: 40% of rams genotyped and 10% incorrect pedigree; hr40y1: 40% of rams genotyped and 1% incorrect genotype; hr40y2: 40% of rams genotyped and 2% incorrect genotype; rand: 5% breeding stock (male and female) genotyped. [file 12711_2024_947_MOESM1_ESM.png]

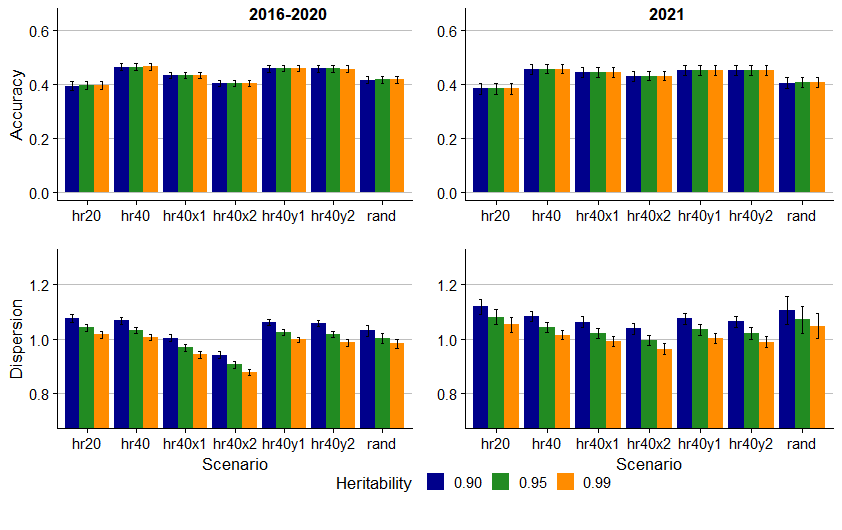

Supplement: Supplementary file 2 — Additional file 2. Accuracy and dispersion bias for predicting the number of copies of the A3 allele in ungenotyped sheep. Error bars represent standard errors across 10 replicates. hr20: 20% of rams genotyped; hr40: 40% of rams genotyped; hr40x1: 40% of rams genotyped and 5% incorrect pedigree; hr40x2: 40% of rams genotyped and 10% incorrect pedigree; hr40y1: 40% of rams genotyped and 1% incorrect genotype; hr40y2: 40% of rams genotyped and 2% incorrect genotype; rand: 5% breeding stock (male and female) genotyped. [file 12711_2024_947_MOESM2_ESM.png]

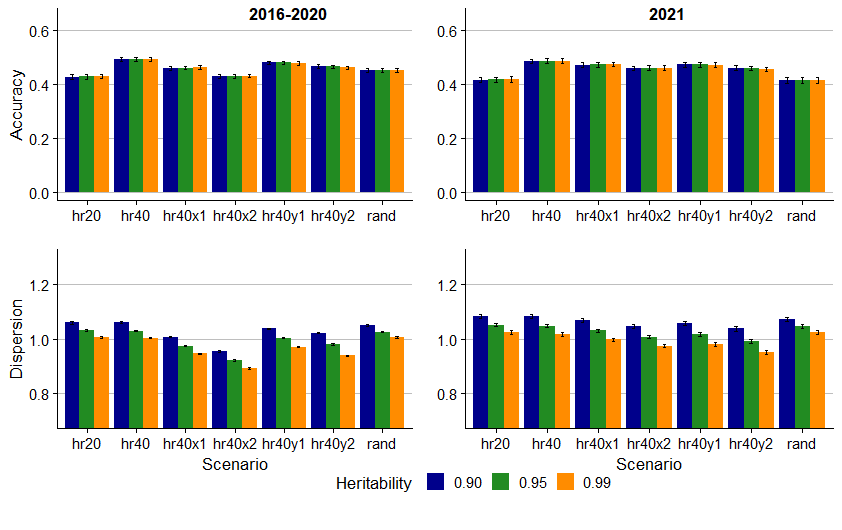

Supplement: Supplementary file 3 — Additional file 3. Accuracy and dispersion bias for predicting the number of copies of the A5 allele in ungenotyped sheep. Error bars represent standard errors across 10 replicates. hr20: 20% of rams genotyped; hr40: 40% of rams genotyped; hr40x1: 40% of rams genotyped and 5% incorrect pedigree; hr40x2: 40% of rams genotyped and 10% incorrect pedigree; hr40y1: 40% of rams genotyped and 1% incorrect genotype; hr40y2: 40% of rams genotyped and 2% incorrect genotype; rand: 5% breeding stock (male and female) genotyped. [file 12711_2024_947_MOESM3_ESM.png]
